# Molecular Insights into the Low Complexity Secreted Venom of *Calliactis polypus*

**DOI:** 10.1093/gbe/evae154

**Published:** 2024-07-17

**Authors:** Hayden L Smith, Daniel A Broszczak, Scott E Bryan, Raymond S Norton, Peter J Prentis

**Affiliations:** School of Biology and Environmental Sciences, Faculty of Science, Queensland University of Technology, Brisbane 4000, Australia; School of Biomedical Sciences, Faculty of Health, Queensland University of Technology, Brisbane 4000, Australia; School of Earth and Atmospheric Sciences, Faculty of Science, Queensland University of Technology, Brisbane 4000, Australia; Medicinal Chemistry, Monash Institute of Pharmaceutical Sciences, Monash University, Parkville, VIC 3052, Australia; ARC Centre for Fragment-Based Design, Monash University, Parkville, VIC 3052, Australia; School of Biology and Environmental Sciences, Faculty of Science, Queensland University of Technology, Brisbane 4000, Australia; Centre for Agriculture and the Bioeconomy, Queensland University of Technology, Brisbane 4000, Australia

**Keywords:** actiniaria, proteomics, peptide toxin, secreted venom

## Abstract

Sea anemones are venomous animals that rely on their venom for prey capture, defense against predators, and intraspecific competition. Currently, comprehensive molecular and evolutionary analyses of the toxin repertoire for sea anemones are limited by a lack of proteomic data for most species. In this study, proteo-transcriptomic analysis was used to expand our knowledge of the proteinaceous components of sea anemone venom by determining the secreted venom proteome of *Calliactis polypus*. Electromechanical stimulation was used to obtain the secreted venom of *C. polypus*. We identified a low complexity proteome that was dominated by toxins with similarity to known neurotoxins, as well as six novel toxin candidates. The novel putative toxin candidates were found to be taxonomically restricted to species from the superfamily Metridioidea. Furthermore, the secreted venom of *C. polypus* had only three putative toxins in common with the venom of acontia from the same species and little similarity with the secreted venom of closely related species. Overall, this demonstrates that regionalized and lineage-specific variability in toxin abundance is common among sea anemone species. Moreover, the limited complexity of the toxin repertoire found in *C. polypus* supports the idea that peptide neurotoxins make up the dominant toxin arsenal found in the venom of sea anemones.

**Significance**

Animal toxins were initially used to develop anti-venom for medical purposes, but over recent decades have garnered interest as candidates for therapeutic development. In this study, we provide a comparative analysis of candidate toxins found in the secreted venom of the sea anemone, *Calliactis polypus*. We found that most toxins were taxonomically restricted and that lineage-specific toxin variation is common among sea anemone species. Overall, we demonstrate the value of using multiomics approaches for future research in toxin pharmacology and the study of toxin family evolution.

## Introduction

Toxin gene expression and protein abundance in various animal lineages have provided important insights into the evolution of toxin gene families (Moran et al. 2008; Sunagar and Moran 2015; Dowell et al. 2016; Surm et al. 2019; Koch et al. 2022). While the evolution of toxin gene families in young animal lineages (e.g., <54 Ma for snakes and cone snails; Sunagar and Moran 2015) is often dominated by positive selection (Juárez et al. 2008; Puillandre et al. 2010; Sunagar and Moran 2015; Phuong and Mahardika 2018), those in older lineages, such as sea anemones, predominantly evolved under negative selection (Sunagar et al. 2013; Sachkova et al. 2014; Jouiaei et al. 2015; Sunagar and Moran 2015; Surm et al. 2019; Lüddecke et al. 2022; E.G. Smith et al. 2023), which maintains the multi-purpose role of their venom in prey capture, defense, and inter- and intra-species competition (Wallace 2008; Sunagar and Moran 2015; Macrander et al. 2016; Prentis et al. 2018). Interestingly, examination of sea anemone venom proteomes has revealed a greater number of toxin families with neurotoxic activity than families with other modes of action (Madio et al. 2017; Ashwood et al. 2022; E.G. Smith et al. 2023). This indicates that the use of paralytic and pain-inducing compounds is important for the survival of this animal group.

Neurotoxins derived from venomous animals can manipulate the activity of electrical signals in nerves and other tissues, typically by altering the activation and inactivation of ion-gated channels (Norton 1991; Kalia et al. 2015; Ahern et al. 2016; Bourinet and Zamponi 2017; Herzig et al. 2020). In sea anemones, neurotoxins active on sodium and potassium channels have been identified in several species (Cariello et al. 1989; Prentis et al. 2018; Madio et al. 2019; Fu et al. 2021; Menezes and Thakur 2022; Monastrynaya et al. 2023; Lara et al. 2023), and putative neurotoxins continue to be discovered and/or characterized (Madio et al. 2018; Sunanda et al. 2018; Elnahriry et al. 2019, 2024; Sachkova et al. 2020a; Krishnarjuna et al. 2021; Ashwood et al. 2023). For example, two ShK-like peptides were identified in *Nematostella vectensis*, with both having a paralytic and lethal activity on zebrafish larvae (Sachkova et al. 2020a). Toxins and toxin families are typically identified using transcriptomics, with different venom arsenals found within each species (Macrander et al. 2015; Rivera-de-Torre et al. 2018; Surm et al. 2019; Mitchell et al. 2020; Ashwood et al. 2021; Delgado et al. 2022), but a lack of proteomic data limits our understanding of whether these genes have been recruited to venom-producing cell types. Furthermore, proteomic studies of secreted venoms in sea anemones have revealed that putative neurotoxin families likely represent the majority of toxins found in their venom, despite transcriptomic data revealing a more diverse potential repertoire (Madio et al. 2017; Ashwood et al. 2022; H.L. Smith et al. 2023). This difference between proteomic and transcriptomic data has been linked to differential expression influenced by biotic and abiotic factors, such as tissue/structure type, developmental stage, bleaching, and salinity (Macrander et al. 2016; Columbus-Shenkar et al. 2018; Madio et al. 2018; Hoepner et al. 2019; Surm et al. 2019; Sachkova et al. 2020b; Ashwood et al. 2021, 2022). Alternatively, this variation could imply that some genes have been incorrectly assigned as toxins or that current techniques fail to capture the full complement of proteins. Therefore, a combination of proteo-transcriptomic methods is necessary to ensure the identification of toxin candidates and ultimately enhance our understanding of toxin evolution.

In this study, we have examined the secreted venom proteome from the sea anemone *Calliactis polypus* to identify characterized and novel toxin families, as well as the selective forces acting on the evolution of novel toxin genes. Using mass spectrometry and bioinformatic approaches, we observed a dominance of neurotoxins, with six novel toxin candidates in the secreted proteome, all with a limited taxonomic distribution among sea anemones. One novel peptide of interest has limited similarity to a sodium channel toxin found in some species of spider, which further supports the theory for the dominance of neurotoxins in the secreted proteome of *C. polypus*. These results improve our understanding of the venom arsenal of *C. polypus* and the evolution of toxins in sea anemones, and they highlight the importance of continued research in elucidating the molecular mechanisms involved in the recruitment of novel toxins in sea anemones.

## Materials and Methods

### Sea Anemone Collection and Venom Milking

*Calliactis polypus* individuals were collected from Frenchman's Beach (Queensland, Australia) and acclimated in an artificial seawater (ASW) system at the Queensland University of Technology, Brisbane, as described previously (H.L. Smith et al. 2023). Anemones were fed artemia and then deprived of food for 3 d before venom was milked using electromechanical stimulation. Specimens were flushed with ASW before salt buffer (500 mM NaCl, 10 mM KCl, 20 mM Na_2_SO_4_, and 2 mM NaHCO_3_) was added to a plastic beaker containing an individual (∼10 mL to cover the entire body). Specimens were then milked for 1 min by stroking the tentacles and column with metal probes attached to an A-M Systems Isolated Pulse Stimulator (Model: 2100) at 10 V with a pulse duration of 1 ms every 10 ms. Venom extracts were transferred to 15-mL LoBind conical tubes and stored at −80 °C before downstream processing. Four biological replicates with two technical replicates of each were acquired.

### Proteome Extraction and Generation

Samples of the secreted venom were freeze-dried using a vacuum cooling system (CHRIST Alpha 1-4 LDplus) at −55 °C and 1 bar, resuspended in 3 mL Milli-Q water, and then desalted using Biotech CE Tubing (MWCO: 100 to 500 Da; Spectrum Laboratories, Inc.); tubing was suspended in Milli-Q water at 4 °C for 15 h with five water changes to remove excess salts. Desalted samples were freeze-dried and resuspended in 100 µL Milli-Q water. Protein and peptide preparation and mass spectrometry analysis were performed as described previously (H.L. Smith et al. 2023) using Data-Dependent Acquisition (DDA) for each sample. Briefly, protein concentration was estimated using A_280_ on a NanoDrop (absorbance 1.0 = 1 mg/mL), and then, approximately 20 µg of total protein for each sample was reduced with dithiothreitol (10 mM) and alkylated with iodoacetamide (40 mM) before tryptic digestion (1:100) overnight at 37 °C. Peptides were desalted using strong cation-exchange resin (Empore) Stage-tips, resuspended with indexed retention time peptides (in 2% acetonitrile, 0.1% formic acid), and subsequent data acquisition was performed using a TripleTOF 5600+ mass spectrometer (Sciex).

### Proteome Annotation

Proteome annotation for the secreted venom of *C. polypus* was performed as described previously (H.L. Smith et al. 2023) using the updated version of TransDecoder (v3) with a minimum length of 30 amino acid residues to ensure detection of small toxin peptides. The resultant fasta file was appended with candidate toxin sequences from our previous study of the acontial proteome of *C. polypus* (H.L. Smith et al. 2023) to identify overlap. This custom database, including sequence data for the common Repository of Adventitious Proteins and indexed retention time peptides, was used in combination with ProteinPilot (v5.0) to search mass spectra for high-confidence peptide matches across all eight replicates to create a single dataset, denoted as the secreted venom of *C. polypus*. Raw data have been deposited to the ProteomeXchange Consortium via the PRIDE partner repository (https://www.ebi.ac.uk/pride/) with the dataset identifier PXD045890.

Label-free quantification analysis was performed on the DDA data for all eight replicates using MS1 Filtering to obtain a relative abundance of the toxin and toxin-like proteins and peptides identified in the secreted venom of *C. polypus*. Skyline (v23.1) was used based on the default settings with the following changes: Peptide Settings—Max Missed Cleavages (2), Min Length (7), Structural Modifications [Carbamidomethyl {C} and Oxidation {M}]; Transition Settings—Precursor Charges (2, 3, 4), Precursor Mass Analyzer (time of flight with 10,000 resolving power). MS1 Filtering data were manually curated to reduce retention time window biases for low-intensity peaks (<1,000) and noise. A box and whisker plot (supplementary fig. S1, Supplementary Material online) was generated based on the peak areas of peptide spectra for all DDA data, with outliers removed and the median excluded for calculations of the quartile values.

### Toxin and Toxin-Like Gene Identification

Candidate toxin and toxin-like genes were determined using methods reported previously for 15 transcriptomes (van der Burg et al. 2016; Surm et al. 2019; H.L. Smith et al. 2023) and an additional four transcriptomes from *Alvinactis* sp. (NCBI Accession PRJNA722768; Xu et al. 2022), *Condylactis gigantea* (NCBI Accession PRJEB21970), *Entacmaea quadricolor* (NCBI Accession PRJEB21970), and *Metridium senile* (NCBI Accession SRR6480802; Praher et al. 2021). Additionally, candidate toxin and toxin-like genes were also analyzed with hmmscan (https://www.ebi.ac.uk/Tools/hmmer/search/hmmscan) for the six available search databases, Pfam, TIGRFAM, Gene3D, Superfamily, PIRSF, and TreeFam. Sequence Read Archive data were obtained from NCBI and assembled using Trinity (v2.13; Haas et al. 2013) as previously described (Prentis and Pavasovic 2014; H.L. Smith et al. 2023). Homologous candidate transcripts for six candidate neurotoxins and six novel toxin candidates found in the secreted venom proteome of *C*. *polypus* were identified in the 19 transcriptomes analyzed. Candidate sequences were subsequently filtered to ensure the presence of signal peptides and significant hits (*P*-value ≤ 0.001) against NCBI (https://blast.ncbi.nlm.nih.gov/Blast.cgi), UniProt (https://www.uniprot.org/) and SMART (http://smart.embl-heidelberg.de/) databases.

### Phylogenetic and Selection Analysis

Phylogenetic and selection analysis using methods established previously (H.L. Smith et al. 2023). Phylogenetic analysis was performed on ShKC10-Cpp1a, calitoxin-Cpp1a, and Na1-Cpp1a, and selection analysis was performed on ShKC10-Cpp1a, the only candidate with a broad distribution and high gene copy numbers in the transcriptome analysis. Alignment of nucleotide and amino acid sequences was performed using MUSCLE (Edgar 2004), with amino acid sequences for the mature proteins used to construct a phylogenetic tree in IQ-TREE (http://iqtree.cibiv.univie.ac.at/; Trifinopoulos et al. 2016) for 10,000 bootstrap alignments, and whole sequences for nucleotides used to perform Fixed Effects Likelihood analysis (100 bootstrap resampling *P*-value ≤ 0.05) via the Datamonkey server (http://www.datamonkey.org/).

## Results

### Secreted Venom Sampling

Electromechanical stimulation of *C. polypus* produced no visible signs of milked venom, which can be seen in some other species, such as the production of a white fluid on the tentacle tips of *Telmatactis stephensoni* (unpublished observations). Tentacle retraction was minimal, enabling a full sweep of the probes over the whole tentacle and body column to ensure maximum discharge of venom.

### Identification of Toxins in the Proteome

**Fig. 1.**
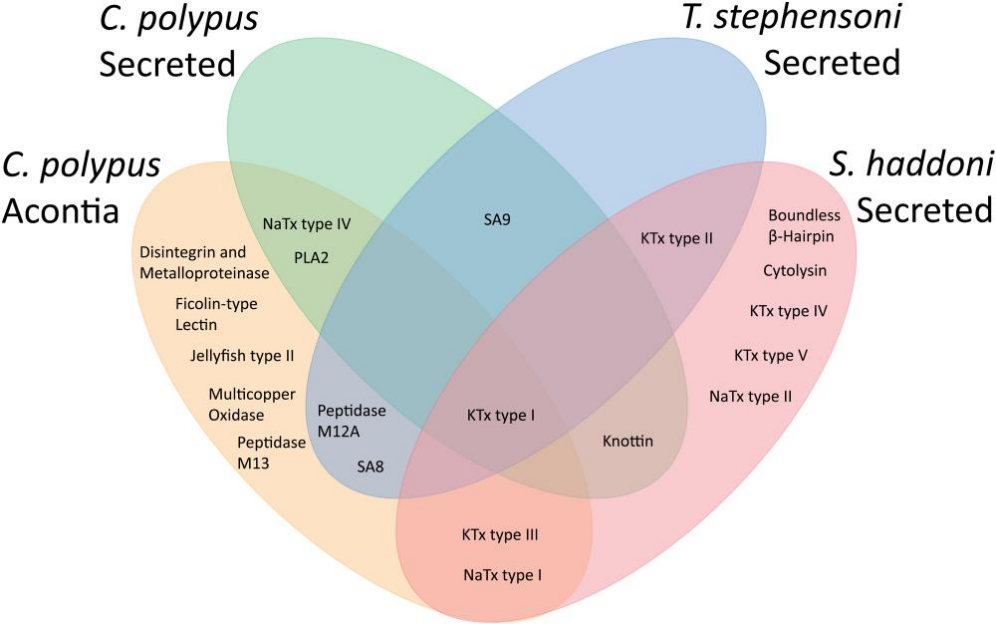
Venn diagram of toxin families found in proteomic data of three sea anemone species, *Calliactis polypus*, *Telmatactis stephensoni,* and *Stichodactyla haddoni*. Data derived from H.L. Smith et al. (2023) (Acontia), Ashwood et al. (2021) and Madio et al. (2019), respectively. Phospholipase A2 toxin (PLA2), potassium channel toxin (KTx), sodium channel toxin (NaTx), sea anemone 8 toxin (SA8), and sea anemone structural class 9 toxin (SA9).

Fifteen toxin and toxin-like proteins and peptides were found in the secreted venom of *C. polypus* (Table 1; supplementary table S1, Supplementary Material online; supplementary table S2, Supplementary Material online). These data further highlight the disparity of toxin profiles for sea anemones, with very few of the toxin families identified in proteomic data present in closely related lineages. Four datasets using similar methodologies for toxin identification had only 50% of the toxin families in common with two or more datasets, and only the potassium channel (KTx) type I toxin (ShK) family is found in all four datasets (Fig. 1). These included nine sequences that were similar to known toxins, with the remaining six having limited or no identity to current toxin or toxin-like peptides. The secreted proteome contains 23.0% of the 65 toxin candidates identified in the transcriptome of *C. polypus* (supplementary table S3, Supplementary Material online). Interestingly, of the nine candidates with similarity to known functions, there are only two candidates (c56806_g1_i1, denoted as PLA2-Cpp1a and c56939_g1_i1) classified in toxin families associated with enzymatic activity, compared with seven candidates associated with neurotoxic activity. Additionally, two neurotoxins, KTx type I (ShK-like) and NaTx (calitoxin), and a putative neurotoxin (transcript c89724_g1_i1, denoted as NaH-Cpp1a) that has sequence similarity to sodium channel inhibitors with the PFAM domain, Toxin_12 (InterPro ID: PF07740; *P*-value = 0.0035), were the only candidates with high-coverage (>90%) for the mature peptide sequence. Overall, these data identified a diversity of neurotoxic peptides and proteins, with a higher copy number for ShK and ShK-like peptides (Table 1; supplementary table S1, Supplementary Material online), indicating a highly specific role for the secreted venom of *C. polypus*.

**Table 1:** Putative toxin candidates with high-confidence (≥95%) peptide matches from the mass spectra

Toxin family	Contig (Identifier)	Whole sequence length	Mature peptide length	Peptide hits (Conf ≥95)	Sequence coverage (%)	Mature peptide coverage (%)
Knottin-like	c51674_g1_i3	85	48	5	21.18	27.69
KTx type I (ShK)	c45616_g1_i1 (ShK-Cpp3a)	95	34	3	24.21	67.65
KTx type I (ShK)	c113611_g1_i1 (ShK-Cpp2a)	79	37	4	27.85	59.46
KTx type I (ShK)	c52694_g1_i1 (ShK-Cpp1a)	84	38	2	17.86	39.47
KTx type I (ShK-like)	c32422_g1_i1 (ShKC10-Cpp1a)	88	69	10	72.73	92.75
NaTx (calitoxin)	c40761_g1_i1 (calitoxin-Cpp1a)	92	48	23	52.17	100.00
Phospholipase A2	c56806_g1_i1 (PLA2-Cpp1a)	162	127	8	38.89	45.00
Phospholipase A2-like	c56939_g1_i1	154	137	4	18.83	21.17
Sea anemone structural class 9	c44591_g1_i1 (SA9-Cpp1a)	117	88	5	35.90	42.86
Unknown C6	c1610_g1_i1	70	41	2	30.00	42.86
Unknown C6	c47813_g1_i1	77	57	6	37.66	50.88
Unknown C6	c47813_g1_i2	77	57	7	38.96	52.63
Unknown C6	c53934_g1_i2	69	48	3	30.43	43.75
Unknown C6	c89724_g1_i1 (NaH-Cpp1a)	73	31	7	38.36	90.32
Unknown C8	c49643_g1_i1	80	61	11	43.75	49.18

Toxin families with an unknown function and/or low homology to known toxins are highlighted in bold, with the number of cysteines (C) listed in the name. Whole sequence coverage refers to whole protein sequence and mature peptide coverage refers to sequence without predicted signal peptide, propeptide, and cleavage sites.

MS1 Filtering analysis for each toxin identified in the whole proteome revealed that three neurotoxins and a functionally unknown peptide encoded by transcript c47813_g1_i2 were the most abundant proteins across the eight replicates obtained for the secreted venom of *C. polypus* (supplementary fig. S1, Supplementary Material online). There was high variability in the peak intensities and abundance estimates among the replicates that suggest differential expression, but more in-depth analyses are needed in order to validate this observation to exclude methodological limitations of the current study. The neurotoxins with the highest relative abundance were two ShK toxins (ShK-Cpp1a and ShK-Cpp2a) and the putative sea anemone structural class 9 (SA9) toxin. These data further support the hypothesis that neurotoxins are the dominant toxin family in most sea anemones.

**Fig. 2.**
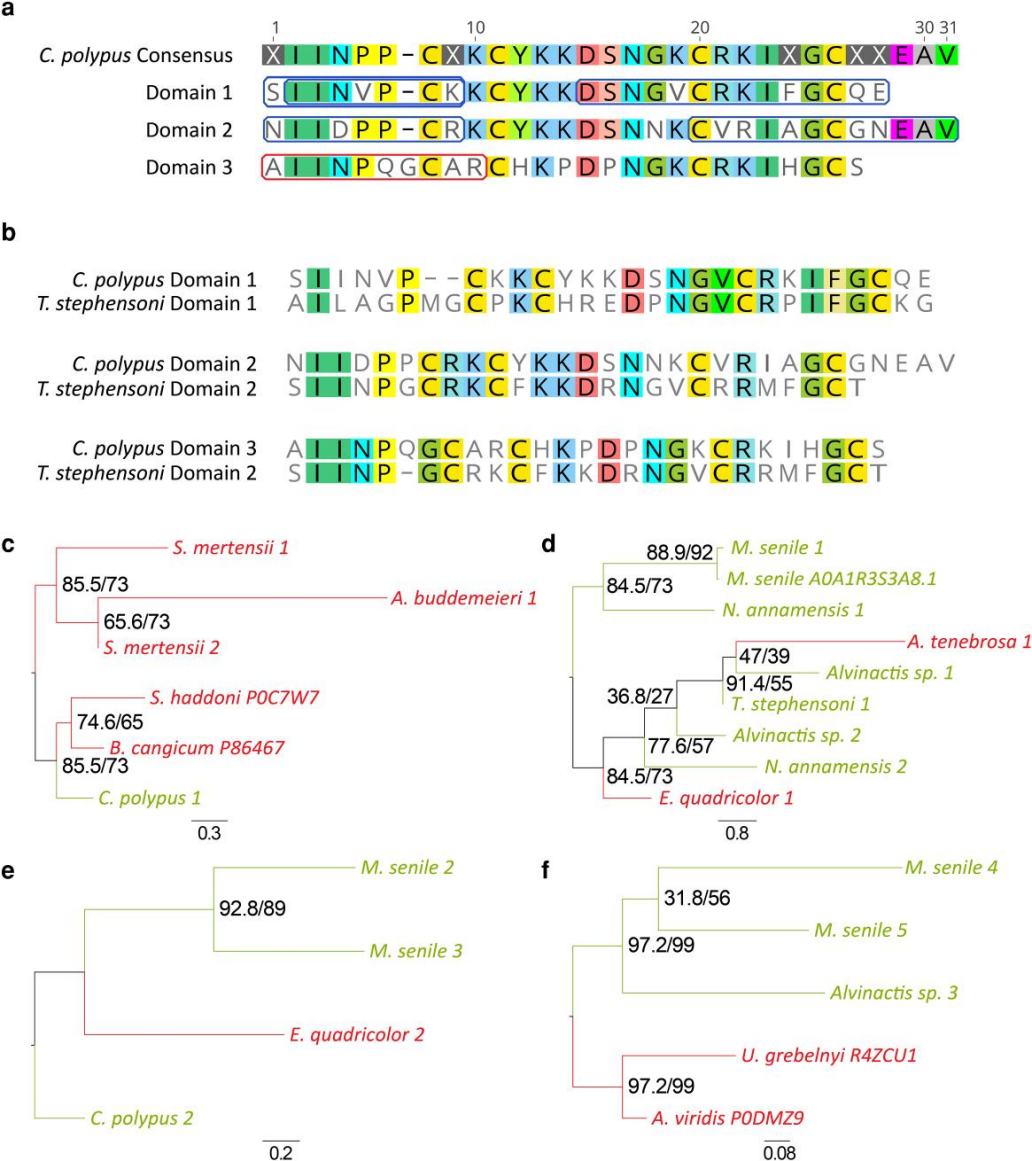
Amino acid sequence alignment of the tandem domains for the SA9 toxin family sequences in *C. polypus* and T*. stephensoni*. a) Alignment of the three tandem domains for SA9-Cpp1a in C. polypus. The five peptide matches from the secreted venom proteome are highlighted with a box. Peptide match highlighted for Domain 3 denotes a confidence of 94.4 (threshold ≥ 95.0). b) Alignment of the tandem domains for SA9-Cpp1a in *C. polypus* with the SA9 toxin identified in the secreted venom of *T. stephensoni* (transcript TR43510|c0_g1_i1|CDS2; Ashwood et al. 2022). Alignment includes two copies of Domain 2 for the *T. stephensoni* sequence to show consensus with Domains 2 and 3 of *C. polypus*. c to f) Maximum-likelihood phylogenetic trees of SA9 candidate toxins in the transcriptomes of actiniarian species based on the number of tandem SA9 domains (one, two, three, and four tandem domains for c, d, e, and f, respectively). Support values shown as approximate likelihood ratio testing (0 to 1)/Maximum-Likelihood bootstrap (0 to 100). Gene transcripts were identified based on their species name followed by count number. Sequences denoted with an alphanumeric code are SA9 toxins from the SwissProt/UniProt databases.

The limited proteomic data and research for the SA9 toxin make transcript c44591_g1_i1 (denoted as SA9-Cpp1a) ideal for current and future studies. The SA9 toxin type is defined by its short 4-cysteine framework domain, typically with multiple tandem peptide domains in a single protein that are separated by the lysine-arginine (KR) cleavage site (Osmakov et al. 2013). The toxin candidate, SA9-Cpp1a, has three tandem peptide domains that are separated by two KR motifs and have sequence similarity to the boundless β-hairpin structure (Osmakov et al. 2013). Alignment of these domains has a consensus sequence of 31 residues (Fig. 2a); 11 are conserved across all domains, 11 are conserved in two domains, and nine residues are mismatches (including glutamic acid-alanine-valine at positions 29 to 31) with one indel at position seven. Interestingly, a didomain protein was found in the secreted venom of *T. stephensoni* (Ashwood et al. 2022), which has approximately 50.0% sequence similarity when aligned to two of the three domains for SA9-Cpp1a (Fig. 2b). As *Telmatactis* and *Calliactis* are closely related genera, this demonstrates that an additional internal duplication event most likely occurred in the *C. polypus* genome after the divergence of these two species, although a domain deletion in *T. stephensoni* is also possible. Across the transcriptomes analyzed, a total of 20 SA9 candidates were found (supplementary table S4, Supplementary Material online). Of these, four, eight, four, three, and one candidates had one, two, three, four, and five tandem domains, respectively. Independent analysis for each tandem domain framework revealed a greater number of sequences for the single domain in Actinioidea, and a greater number for the double domain in Metridioidea (Fig. 2c to f). The single- and double-domain frameworks had more sequences compared with the triple and quadruple domains. These data suggest lineage-specific evolution of the SA9 toxin family within sea anemone species.

### Sodium Channel Neurotoxin Candidate

Only a single sodium channel toxin candidate (transcript c40761_g1_i1, denoted as calitoxin-Cpp1a) was identified in the secreted venom of *C*. *polypus*. This candidate has 66.66% similarity (for the mature peptide) to the only two characterized calitoxins, which are both found in a species of the same genus, *C. parasitica*.

### Potassium Channel Neurotoxin Candidates

Four ShK candidate peptides were found in the secreted venom of *C. polypus*. Transcript c52694_g1_i1 (denoted as ShK-Cpp1a) is moderately similar (∼50% for the mature peptide) to other ShK toxins in SwissProt (i.e. Kappa-actitoxin-Aer3a, *P*-value 7.9e10^−11^; Hasegawa et al. 2006) and shows 54.76% sequence similarity with an ShK peptide found previously in the secreted venom of *T. stephensoni* (Ashwood et al. 2022; Sanches et al. 2024). Transcript c113611_g1_i1 (denoted as ShK-Cpp2a), has partial coverage with moderate similarity to nematode metalloendopeptidases (i.e. UniProt ID A0A368H313; *P*-value: 2.2e^−5^) and lacked significant matches (*P*-value ≤ 1.0e^−6^) in the transcriptomic data of other sea anemone species. Transcript c45616_g1_i1 (denoted as ShK-Cpp3a) has no similarity to any sequence in the NCBI and UniProt databases, and no homologous sequences were found in the transcriptomes analyzed. Conversely, transcriptomic analysis revealed that ShKC10-Cpp1a has up to three gene copies in six species and is found predominantly in Metridioidea (five species vs. only one in Actinioidea; supplementary table S5, Supplementary Material online). This transcript has a didomain structure with a 4-cysteine domain followed by a ShK-like 6-cysteine domain and has multiple peptide matches across the didomain structure. As ShK peptides contain six cysteine residues (Shafee et al. 2019; Tudor et al. 1996), it is more likely that the protein has a single domain structure with a 10-cysteine framework. While ShKC10-Cpp1a has a similarity to ShK sequences, which could be indicative of a neurotoxic activity, it is possible that the 10-cysteine framework has an alternative function or mode of action. Notably, this candidate was found in the acontia venom (H.L. Smith et al. 2023), which further supports the likelihood of a toxin activity.

**Fig. 3.**
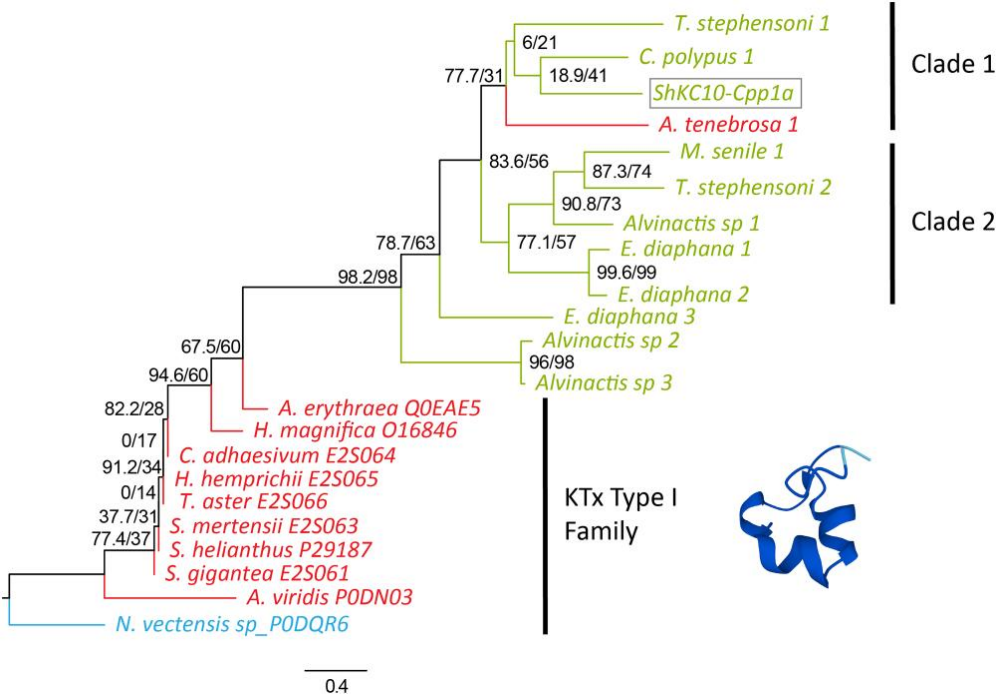
Maximum-likelihood phylogenetic tree of the 10-cysteine ShK-like sequences in the transcriptomes of actiniarian species. Sequence found in the proteome of secreted venom for *C. polypus* (ShKC10-Cpp1a) is indicated with a box. Support values shown as approximate likelihood ratio testing (0 to 1)/Maximum-likelihood bootstrap (0 to 100). Gene transcripts were identified based on their species name followed by count number. Sequences denoted with an alphanumeric code are potassium channel toxins from the SwissProt database. Inset image is the derived AlphaFold structure for P29187.

Phylogenetic analysis of ShKC10-Cpp1a revealed moderate clustering of candidate sequences that resolve outside the single domain ShK sequences from SwissProt (Fig. 3). Clade 1, which contains this toxin candidate, sits outside the Metridioidea-specific cluster, Clade 2, with low-moderate support. The grouping of species-specific duplicates in *C*. *polypus*, *Alvinactis* sp., and *Exaiptasia diaphana* are suggestive of independent duplication events or extensive rounds of gene loss throughout Actiniaria, except for select species of Metridioidea. Selection analysis of ShKC10-Cpp1a found evidence of purifying selection at 20 of 79 sites (*P*-value ≤ 0.05) in the mature peptide sequence (supplementary table S6, Supplementary Material online), further supporting the likelihood that this protein has a functional role in the secreted proteome. The prominence of neutral evolution at most sites and the low signal intensity found in phylogenetic analysis likely indicate a lack of sequence conservation between these sequences.

### Undescribed Toxin Candidates

Six candidates from the secreted venom proteome are currently undescribed and may have novel structures and functions (supplementary table S7, Supplementary Material online). Two candidates have hits in the PFAM and UniProt databases: NaH-Cpp1a has a similarity to the Toxin_12 domain (InterPro ID: PF07740; *P*-value = 0.0035) and c49643_g1_i1 has a similarity to an uncharacterized protein (A0A6P8IHZ2, *P*-value = 7.9e^−03^), respectively. The other four candidates have no similarity to any known sequence in any of the databases searched, but all six have ≤ 80 amino acids (and likely mature peptides between 31 and 61 residues), a signal peptide, and a cysteine-rich framework, suggestive of a toxin function. Peptide matches for these candidates have a coverage range of between 40% and 90% for the mature peptide sequence (Table 1). Of note, only NaH-Cpp1a was analyzed in-depth owing to the limited sequence identities for the other candidates. The mature peptide coverage of NaH-Cpp1a has the highest coverage among the unknown candidates, at 90.32% and 31 amino acids, and has the third highest coverage in the secreted proteome. Furthermore, NaH-Cpp1a has a cysteine framework (CX_7_-CX_6_-CCX_4_-CX_6_-CX_3_; where X_n_ is the number of amino acids) similar to those of known neurotoxins in spiders (hainantoxin-III and theraphotoxin-Hhn2b; *P*-value = 0.018; Xiao and Liang 2003; Tang et al. 2010; Liu et al. 2013; supplementary fig. S2, Supplementary Material online). However, we observed a limited distribution of these six putative toxins in other sea anemone species (supplementary table S7, Supplementary Material online). The best candidate for toxin activity, NaH-Cpp1a, has three additional homologous sequences in *C. polypus* and only a single homologous sequence in *M. senile* and *Nemanthus annamensis*. Overall, the six novel toxin candidates identified in *C. polypus* are likely to be lineage-specific to the superfamily Metridioidea, with a limited expansion or an extensive loss in the sea anemone species analysed in this study.

### Comparison of the Secreted and Acontial Venoms

**Fig. 4.**
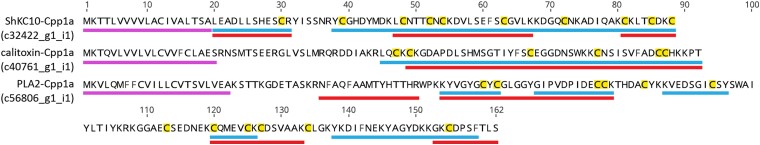
LC-MS/MS sequence coverage for the three putative toxins found in the secreted venom and acontia of *C. polypus*. Cysteine residues highlighted in sequences. Signal peptides are denoted with the first underline of each sequence, coverage of peptide matches in secreted venom is denoted with underlining on first line of each sequence, and coverage of peptide matches in acontia venom is denoted with underlining on second line of each sequence. Coverage of peptide matches is shown as contiguous and overlapping matches, not for individual peptide matches.

Comparisons with published data for the acontia venom proteome in *C. polypus* (H.L. Smith et al. 2023) revealed just three out of 33 proteins in common between the acontia and secreted venoms. There is a distinct variation in the peptide coverage for the three putative toxins found in both data sets (Table 2; Fig. 4), which may relate to the relative abundance of each protein in each sample. There is approximately a 10% increase (100% vs. 91.67%) in mature peptide coverage for calitoxin-Cpp1a and about a 30% increase (92.75% vs. 59.42%) for the ShK-like peptide (c32422_g1_i1, denoted as ShKC10-Cpp1a) in the secreted venom versus the acontia venom. Conversely, there is about a 15% decrease (42.86% vs. 57.14%) in mature peptide coverage for PLA2-Cpp1a. An interesting observation of the secreted venom is the absence of the highly abundant NaTx type I toxin and the high-coverage putative toxins, sea anemone 8 and Unknown 12C, which were found in the acontia venom. Additionally, the presence of the SA9 toxin, as well as multiple copies of ShK and functionally uncharacterized putative toxins found in the secreted venom, highlights a possible broad activity for these neurotoxins.

**Table 2:** Comparison of peptide matches for three putative toxins in the secreted venom and acontia of *C. polypus*

Toxin family	Contig (Identifier)	Secreted venom			Acontia venom		
		Peptide hits (Conf ≥95)	Whole sequence coverage (%)	Mature peptide coverage (%)	Peptide hits (Conf ≥95)	Whole sequence coverage (%)	Mature peptide coverage (%)
KTx type I (ShK-like)	c32422_g1_i1 (ShKC10-Cpp1a)	10	72.73	92.75	4	46.59	59.42
NaTx (calitoxin)	c40761_g1_i1 (calitoxin-Cpp1a)	23	52.17	100.00	4	47.83	91.67
Phospholipase A2	c56806_g1_i1 (PLA2-Cpp1a)	8	37.04	42.86	7	49.38	57.14

Whole sequence coverage refers to whole protein sequence, and mature peptide coverage refers to sequence without predicted signal peptide, propeptide, and cleavage sites. Acontia data retrieved from previously published material (H.L. Smith et al. 2023) with addendum to incorrect value reported for transcript c40761_g1_i1 sequence coverage % (supplementary table S3, Supplementary Material online).

**Fig. 5.**
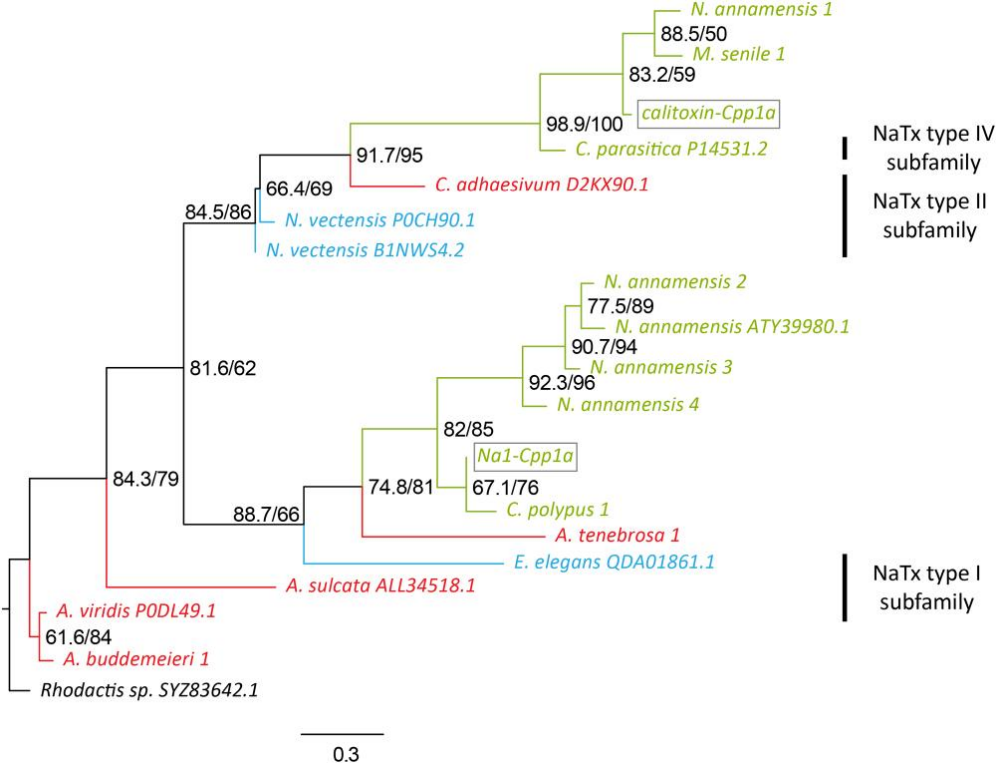
Maximum-likelihood phylogenetic tree of two candidate NaTx sequences (calitoxin/type IV and type I families) in the transcriptomes of actiniarian species and the outgroup, *Rhodactis* sp. from order Corallimorpharia. Sequences found in the venom proteomes for *C. polypus*, calitoxin-Cpp1a (calitoxin; secreted and acontia) and Na1-Cpp1a (NaTx type I; acontia), are indicated with a box. Toxin subfamilies for published sequences reported as per UniProt and Monastrynaya et al. (2023). Support values shown as approximate likelihood ratio testing (0 to 1)/Maximum-Likelihood bootstrap (0 to 100). Gene transcripts were identified based on their species name followed by count number. Sequences denoted with an alphanumeric code are sodium channel toxins from the SwissProt/UniProt databases.

Phylogenetic analysis of transcript c50240_g1_i1 (denoted as Na1-Cpp1a) from the acontia venom of *C. polypus* (H.L. Smith et al. 2023) and calitoxin-Cpp1a revealed strong clustering of candidate sequences that resolve into NaTx subfamily types I and IV, respectively (Fig. 5). Moderate support was observed for the type I and type II cluster, whereas very strong support between type IV and type II suggests these toxin types are more closely related. There are clear groupings of each NaTx type into each taxonomic superfamily of sea anemones, despite a lack of sequences found in non-Metridioidea lineages. Furthermore, two and four homologous sequences to Na1-Cpp1a were observed in *C*. *polypus* and *N. annamensis*, respectively, which indicates multiple duplication events in select species or extensive rounds of gene loss throughout most sea anemone lineages.

## Discussion

It has been hypothesized that evolutionary novelty in animal venom is key to the success of venomous species (Moran et al. 2008; Casewell et al. 2013; Sunagar and Moran 2015; Dowell et al. 2016; Koch et al. 2022). Sea anemones possess a multi-purpose venom that is required for prey capture and digestion, defense against predators, and inter- and intra-species competition (Wallace 2008; Macrander et al. 2016). As the study of toxins in sea anemones expands, we uncover new and novel toxin candidates (Madio et al. 2017; H.L. Smith et al. 2023) and improve our understanding of toxin family evolution in this unique venomous lineage. In this study, we identified six uncharacterized toxin candidates in the secreted venom of *C. polypus*, as well as a vastly different toxin repertoire compared with other sea anemone species and the acontia-derived venom of the same species.

### Variation in Venom Abundance

Venom in sea anemone species has a multi-purpose role that changes its abundance across different tissues or structures (Wallace 2008; Macrander et al. 2016; Ashwood et al. 2021). Our data from the *C*. *polypus* secreted venom, in combination with the venom from acontia, demonstrate marked differences in venom composition. Each has a limited number of toxin families present, with the secreted venom having reduced complexity but a greater variation in copy number compared with acontia venom. Of note, the NaTx type I toxin that exhibited a high relative abundance in the acontia venom was not detectable in the secreted venom, further supporting the idea that the acontia is a specialised structure with a unique role in predator deterrence (Ross 1971; McLean and Mariscal 1973; H.L. Smith et al. 2023). Furthermore, the NaTx type I toxin had at least a 10-fold greater abundance compared with other toxins in the acontia venom (H.L. Smith et al. 2023), whereas, in the secreted venom, there are four toxins with higher relative abundances than the other toxins present. The differences observed in relative abundances between the replicates are suggestive of biological variation, which contrasts strongly with the lack of biological variation reported in acontia venom (H.L. Smith et al. 2023). It is also important to note in both the acontia and secreted venoms of *C. polypus*, neurotoxins represent the most abundant toxin family, which is a trend observed in other sea anemone species (Madio et al. 2017; Ashwood et al. 2022). The three putative toxins in common between the secreted and acontia venom are a phospholipase A2, the only known enzymatic toxin in the secreted venom of *C*. *polypus*, calitoxin, a neurotoxin that targets sodium channels in crustaceans (Cariello et al. 1989), and an ShK-like sequence. Thus, we observe a distinct variation in the venom composition between different morphological regions used for venom delivery in a single species, although more work is needed to compare and contrast against different species.

The secreted venom of *C. polypus* has little similarity to the secreted venom of closely- and distantly related sea anemone species (Madio et al. 2017; Mitchell et al. 2020; Ashwood et al. 2022). For example, there are only two toxins in common for the secreted venoms of both *C*. *polypus* and *T*. *stephensoni*, the most closely related species with proteomic data available for its venom (Ashwood et al. 2022). Comparative transcriptomic data also revealed a limited number of sequences with similarity to calitoxin, which suggests this toxin family is restricted to the superfamily Metridioidea, with three species having a single copy and *C. parasitica* having two copies (Spagnuolo et al. 1994). The presence of calitoxin in the secreted and acontia venoms of *C. polypus* highlights the need to elucidate its expression in other tissues and structures of sea anemone species. In particular, whether calitoxin has been recruited throughout the body plan of *Calliactis* species and if it has a broader functional role than just for defense (Ross 1971; McLean and Mariscal 1973). Overall, we observe significant variation in the abundance of toxin families across different morphological structures in *C*. *polypus* but also a very limited overlap of toxins among sea anemone species.

The limited taxonomic distribution of several toxins found in *Calliactis* species could be related to differences in its ecology compared with other closely related species and/or phylogenetic inertia. *Calliactis* species form mutually beneficial relationships with hermit crabs, with the sea anemone providing protection via its venom (Ross 1971; McLean and Mariscal 1973) and likely gaining various biological and environmental benefits (Brooks and Gwaltney 1993; Mercier and Hamel 2008; Mercier et al. 2011; Gusmão et al. 2020). Of the sea anemones we sampled, *N. annamensis* is the most closely related species and has the most toxin candidates in common with *C. polypus*; however, it is ecologically differentiated and forms colonial groups attached to black coral (Excoffon et al. 2009; Gress and Kaimuddin 2021) rather than a symbiotic relationship. Currently, we lack sufficient evidence to strongly support either ecological differences or phylogenetic inertia for toxin turnover, and testing the influence of symbiosis on toxin evolution more robustly would require dense sampling of closely related species with differences in their ecology.

### Neurotoxin Specialization in Secreted Venom

Neurotoxin candidates encompass the majority of toxin and toxin-like peptides in the secreted venom of *C*. *polypus*. There are at least six putative neurotoxins in the secreted venom proteome, representing 40% of the identifiable peptides and proteins in the venom, with another four neurotoxin candidates that increase this value to 66.66%. The high proportion of neurotoxins suggests a need for a diverse range of toxin activities against ion channel targets, which is typically seen in other venomous lineages (Smith et al. 2013; Holding et al. 2016; Calvete 2017; Lüddecke et al. 2022). The secreted venom of *C. polypus* follows a similar pattern to the acontia, producing a low complexity venom dominated by a small number of neurotoxins.

The repertoire of putative neurotoxins in the secreted venom of *C. polypus* is dominated by potassium and sodium channel toxin families. In particular, there are three peptides with ShK motifs, which is a well-studied fold with several representatives that inhibit voltage-gated potassium channels (Prentis et al. 2018; Shafee et al. 2019; Sanches et al. 2024), one peptide with strong similarity to the sodium channel toxin, calitoxin (Cariello et al. 1989), and one peptide with similarity to ion channel toxins associated with variable functions, SA9 (Zaharenko et al. 2008; Osmakov et al. 2013). Of the three ShKs reported, ShK-Cpp1a and ShK-Cpp2a have the expected cysteine framework and a tandem lysine and tyrosine residue between the third and fourth cysteine residues, which is known to be strongly associated with potassium channel inhibition for ShK toxins (Rauer et al. 1999; Murray et al. 2015; Sanches et al. 2024) we note that a ShK-like candidate from the nematocysts of *N. vectensis* exhibited neurotoxic activity in zebrafish larvae despite lacking the lysine-tyrosine motif (Sachkova et al. 2020a). These two ShK toxin candidates also had a higher relative abundance compared with the other two ShK candidates (ShK-Cpp3a and ShKC10-Cpp1a) which lack the tandem lysine and tyrosine residues, suggesting that in *C. polypus* this motif is functionally important. Similarly, calitoxin has been shown to modulate sodium channels in crustaceans (Cariello et al. 1989), although there is limited research on this toxin. Calitoxin is also taxonomically restricted, with similar sequences found in only a few closely related species, giving credence to the possibility that the multiple toxin candidates identified in this study with few or no homologous sequences in other sea anemones are in fact toxins. Ultimately, this study highlights the need for further research to gain a better understanding of the structure, function, and evolutionary significance of novel neurotoxins in sea anemones.

### Novel Peptides Underlie Need for Focused Research

Gaining molecular insights into novel toxin peptides and proteins is vital for future research into structure-function relationships, and the candidates identified in this study present an ideal opportunity. One putative toxin of interest, NaH-Cpp1a, has similarity to the Toxin_12 domain with six cysteines and a short mature peptide sequence of 31 residues (Peng et al. 2002; Liu et al. 2003, 2013), which are characteristic of neurotoxins. Interestingly, its amino acid sequence is closest to hainantoxin-III, a potent sodium channel neurotoxin found in some spider species (Xiao and Liang 2003; Liu et al. 2013), which further highlights the lack of data for toxins in sea anemones. Hainantoxin-III has been shown to target Na_V_1.1, 1.2, 1.3, and 1.7 channels in HEK 293 cells and is thought to have an effect on vertebrate species (Liu et al. 2013). Furthermore, the six unknown proteins, including NaH-Cpp1a, identified in this study are notably absent in the acontia venom of *C. polypus* (H.L. Smith et al. 2023), and there have been no sequences with similarity to these candidate toxins reported in any proteomic data for sea anemone venom (Moran et al. 2013; Madio et al. 2017; Surm et al. 2019; Mitchell et al. 2020; Sachkova et al. 2020a; Ashwood et al. 2022). This is unsurprising given the limited taxonomic distribution of many of the putative toxins identified in this study. In fact, only a single toxin candidate, ShKC10-Cpp1a, had the required distribution and copy number needed to perform selection analysis. This analysis showed that negative selection was acting on multiple codons in this gene, a pattern observed in most sea anemone neurotoxins examined to date (Jouiaei et al. 2015; Sunagar and Moran 2015; Surm et al. 2019). All the other candidate sequences had limited distributions and low copy numbers among the sea anemones analysed, which precludes the analyses needed to determine the molecular mechanisms involved in the evolution of these genes. However, this limited distribution does suggest that these putative toxins could have a highly specific function, with a strong likelihood they are related to the three core roles attributed to sea anemone venom, namely prey capture, defense and competition.

## Supplementary Material

evae154_Supplementary_Data

## Data Availability

Raw data have been deposited to the ProteomeXchange Consortium via the PRIDE partner repository (https://www.ebi.ac.uk/pride/) with the dataset identifier PXD045890.
