# Supplementary material for: Molecular Insights into the Low Complexity Secreted Venom of Calliactis polypus
Source: Genome Biol Evol. 2024 Jul 17;16(8):evae154. doi: 10.1093/gbe/evae154 (PMC11299110; doi:10.1093/gbe/evae154)
Supplement: evae154_Supplementary_Data [file evae154_supplementary_data.docx]

**Molecular insights into the low complexity secreted venom of *Calliactis polypus***

**Hayden L. Smith ^1^, Daniel A. Broszczak ^2^, Scott E. Bryan ^3^, Raymond S. Norton ^4,5^, Peter J. Prentis ^1,6,*^**

1 School of Biology and Environmental Sciences, Faculty of Science, Queensland University of Technology, Brisbane 4000, Australia

2 School of Biomedical Sciences, Faculty of Health, Queensland University of Technology, Brisbane 4000, Australia

3 School of Earth and Atmospheric Sciences, Faculty of Science, Queensland University of Technology, Brisbane 4000, Australia

4 Medicinal Chemistry, Monash Institute of Pharmaceutical Sciences, Monash University, Parkville, Victoria 3052, Australia

5 ARC Centre for Fragment-Based Design, Monash University, Parkville, Victoria, 3052, Australia

6 Centre for Agriculture and the Bioeconomy, Queensland University of Technology, Brisbane 4000, Australia

* Corresponding Author: p.prentis@qut.edu.au


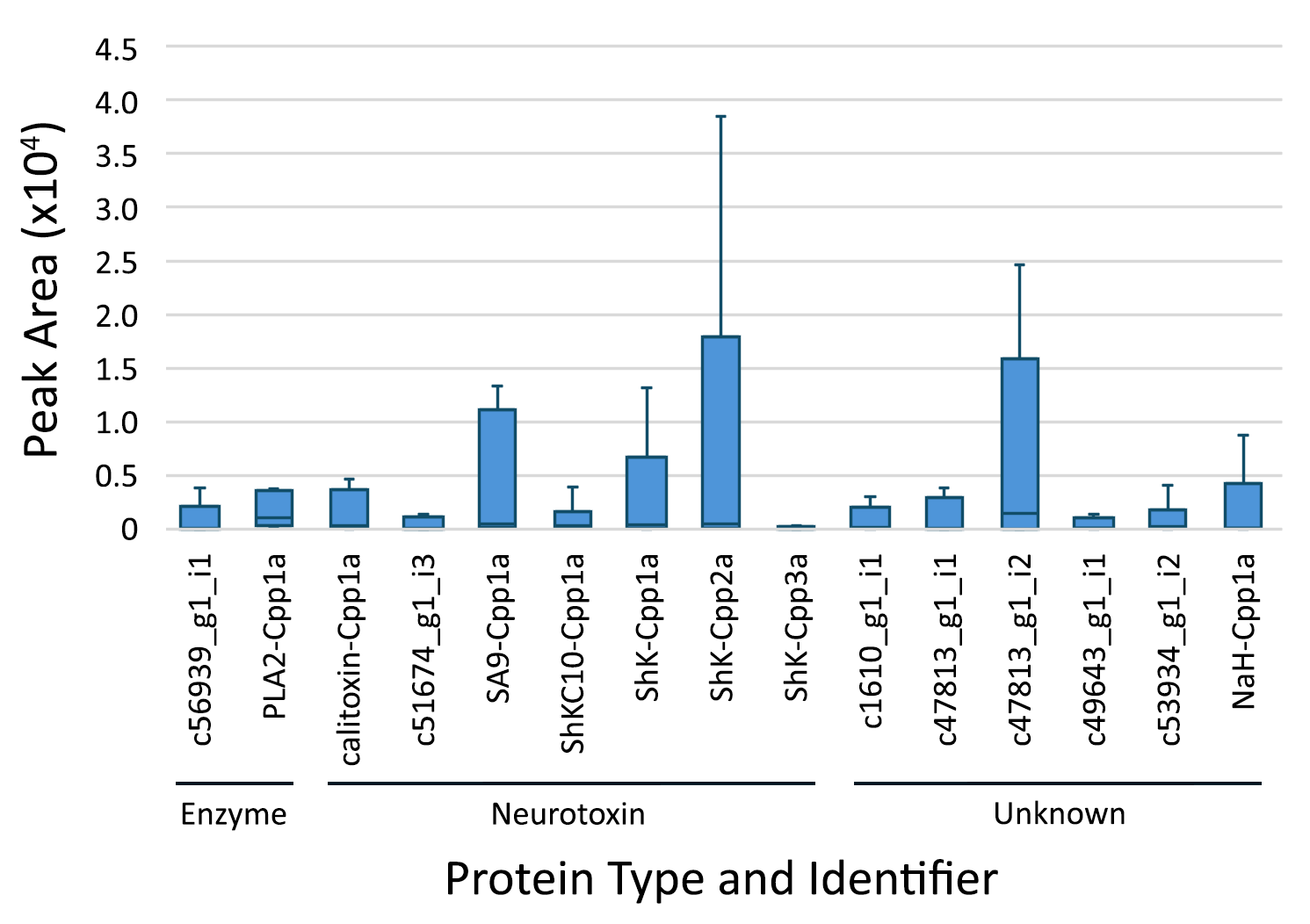


Supplementary Figure 1: Box and Whisker plot for the relative abundances of each toxin and toxin-like candidate identified in the proteome of *Calliactis polypus*. Protein identifiers based on contig name as reported in Table 1 and Supplementary Table 1.


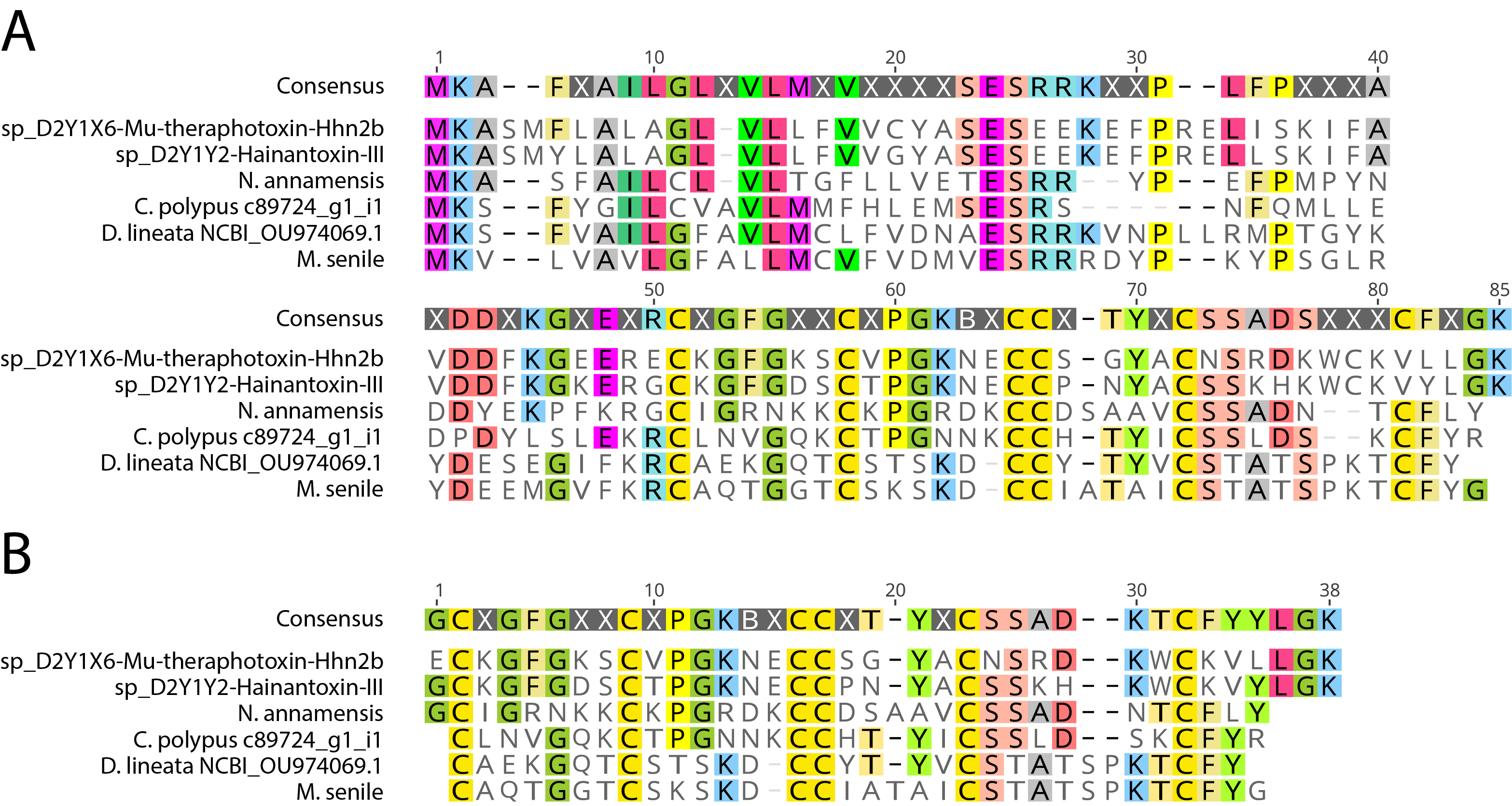


Supplementary Figure 2: Amino acid sequence alignment of transcript c89724_g1_i1 in *C. polypus* and sequences with similarity to sea anemones and two spider toxins with the Toxin_12 domain. (**A**) Alignment of whole sequences. (**B**) Alignment of sequences for the predicted mature peptides. Prefixes with “sp” taken from the SwissProt Database and prefix with “NCBI” taken from the NCBI Database. All other sequences identified in the transcriptomes analysed.

Supplementary Table 1: Annotations for the 15 toxin candidates identified in the secreted venom of *Calliactis polypus* using the NCBI, UniProt and SMART databases. Hit descriptions from each database reported as the most appropriate hit with the highest score.

| Toxin Family | Contig | Peptide Hits (Conf ≥95) | Sequence coverage (%) | Mature Peptide coverage (%) | Blast Accession | Blast Descriptor | Blast e-value | UniProt Accession | UniProt Descriptor | UniProt e-value | SMART Hit | SMART e-value |
| --- | --- | --- | --- | --- | --- | --- | --- | --- | --- | --- | --- | --- |
| Knottin-like | c51674_g1_i3 | 5 | 21.18 | 27.69 | Not significant | uncharacterized protein | 2.00E-19 | A0A6P8IPH9 | Uncharacterized protein (**Unreviewed**) | 2.40E-23 | Pfam: Toxin_35 | 0.02 |
| KTx type I (ShK) | c45616_g1_i1 | 3 | 24.21 | 67.65 | No Similarity | **N/A** |  | No Similarity | **N/A** |  | Pfam: ShK | 1.60E-05 |
| KTx type I (ShK) | c113611_g1_i1 | 4 | 27.85 | 59.46 | Not significant | uncharacterized protein | 0.003 | A0A368H313 | Metalloendopeptidase (**Unreviewed, Low Coverage**) | 0.000022 | Pfam: ShK | 0.0000044 |
| KTx type I (ShK) | c52694_g1_i1 | 2 | 17.86 | 39.47 | Q0EAE5.1 | Kappa-actitoxin-Aer3a | 2.00E-07 | Q0EAE5 | Sea anemone type 1 potassium channel toxin (Type 1a) | 7.50E-11 | Pfam: ShK | 0.0022 |
| KTx type I (ShK-like) | c32422_g1_i1 | 10 | 72.73 | 92.75 | Not significant | uncharacterized protein | 5.00E-09 | A0A6P8IJB3 | Uncharacterized protein (**Unreviewed**) | 1.00E-07 | 2x Pfam: ShK | 0.19 \| 0.014 |
| NaTx (calitoxin) | c40761_g1_i1 | 23 | 52.17 | 100.00 | 2006280B | Calitoxin | 5.00E-33 | P49127 | Sea anemone sodium channel inhibitory toxin | 6.30E-41 | PFAM: Toxin_4 | 1.40E-12 |
| Phospholipase A2 | c56806_g1_i1 | 8 | 38.89 | 45.00 | XP_031552196.1 | Phospholipase A2 | 7.00E-31 | A0A6P8H9J5 | Phospholipase A2 | 4.30E-37 | PA2c | 3.66E-27 |
| PLA2-like (C10) | c56939_g1_i1 | 4 | 18.83 | 21.17 | XP_020914887.1 | Phospholipase A2 | 1.00E-15 | A0A6P8HXC0 | Phospholipase A2 isozyme-like | 1.00E-16 | Pfam: Phospholip_A2_2 | 1.30E-10 |
| Sea Anemone 9 | c44591_g1_i1 | 5 | 35.90 | 42.86 | P0DMZ8.1 | U-actitoxin-Avd13a/b | 7.00E-09 | P0DMZ9 | Sea anemone structural class 9a | 3.10E-14 | **N/A** |  |
| Unknown C6 | c1610_g1_i1 | 2 | 30.00 | 42.86 | No Similarity | **N/A** |  | No Similarity | **N/A** |  | **N/A** |  |
| Unknown C6 | c47813_g1_i1 | 6 | 37.66 | 50.88 | No Similarity | **N/A** |  | No Similarity | **N/A** |  | **N/A** |  |
| Unknown C6 | c47813_g1_i2 | 7 | 38.96 | 52.63 | No Similarity | **N/A** |  | No Similarity | **N/A** |  | **N/A** |  |
| Unknown C6 | c53934_g1_i2 | 3 | 30.43 | 43.75 | No Similarity | **N/A** |  | No Similarity | **N/A** |  | **N/A** |  |
| Unknown C6 | c89724_g1_i1 | 7 | 38.36 | 90.32 | No Similarity | **N/A** |  | D2Y1Z8 | Hainantoxin-III | 1.80E-02 | Pfam: Toxin_12 | 3.50E-03 |
| Unknown C8 | c49643_g1_i1 | 11 | 43.75 | 49.18 | No Similarity | **N/A** |  | A0A6P8IHZ2 | Uncharacterized protein (**Unreviewed**) | 7.90E-03 | **N/A** |  |

Supplementary Table 2: Peptide matches as reported from ProteinPilot (v5.0) for the secreted venom of *Calliactis polypus*. Sequences for peptide matches reported with fullstops ‘.’ To denote the cleavage site.

| Contig | Toxin Family | Peptide Match (Conf ≥95) | Peptide Match (Conf ≥60) [Conf] | Protein Sequence |
| --- | --- | --- | --- | --- |
| c1610_g1_i1 | Unknown C6 | R.GDCPPKGQCQDLR.E |  | MFTAMIFVLLVASYTSHSAEAKYLELIERGDCPPKGQCQDLRERCHWNSDPKSEVCKKMERCGCYGLTLN |
|  |  | R.CHWNSDPK.S |  |  |
| c32422_g1_i1 | KTx Type I (ShK) | K.CKLTCDKC | LEADLLSHE.S [77.7] | MKTTLVVVVLACIVALTSALEADLLSHESCRYISSNRYCGHDYMDKLCNTTCNCKDVLSEFSCGVLKKDGQCNKADIQAKCKLTCDKC |
|  |  | K.DGQCNKADIQAK.C |  |  |
|  |  | K.DVLSEFSCGVLK.K |  |  |
|  |  | K.DVLSEFSCGVLKK.D |  |  |
|  |  | LEADLLSHESC.R |  |  |
|  |  | LEADLLSHESCR.Y |  |  |
|  |  | LEADLLSHESCRY.I |  |  |
|  |  | R.YCGHDYMDK.L |  |  |
|  |  | K.LCNTTCNCK.D |  |  |
|  |  | C.NCKDVLSEF.S |  |  |
| c40761_g1_i1 | NaTx type IV (calitoxin) | 23 peptides (see RAW data, PXD045890) |  | MKTQVLVVLVLCVVFCLAESRNSMTSEERGLVSLMRQRDDIAKRLQCKCKGDAPDLSHMSGTIYFSCEGGDNSWKKCNSISVFADCCHKKPT |
|  |  |  |  |  |
|  |  |  |  |  |
|  |  |  |  |  |
|  |  |  |  |  |
| c44591_g1_i1 | Structural toxin peptide (sea anemone type 9a) | K.CVRIAGCGNEAV.K | R.AIINPQGCAR.C [64.4] | MKTIIAIFSLAAMIVLVRPTPLENDEWTRSIINVPCKKCYKKDSNGVCRKIFGCQEKRNIIDPPCRKCYKKDSNNKCVRIAGCGNEAVKRAIINPQGCARCHKPDPNGKCRKIHGCS |
|  |  | K.DSNGVCRKIFGCQE.K | R.AIINPQGCARC.H [93.1] |  |
|  |  | S.IINVPCK.K | R.SIINVPCKK.C [93.1] |  |
|  |  | R.NIIDPPCR.K | K.CVRIAGCGNEA.V [84.3] |  |
|  |  | R.SIINVPCK.K | I.INPQGCAR.C [84.3] |  |
|  |  |  | K.CYKKDSNGVCR.K [77.2] |  |
|  |  |  | R.KIFGCQE.K [69.1] |  |
|  |  |  |  |  |
|  |  |  |  |  |
|  |  |  |  |  |
|  |  |  |  |  |
| c45616_g1_i1 | KTx type I (ShK) | R.CSDAQSDCAHR.K | K.VKNVYCK.K [91.9] | MKFQLLVVLLLCALCSLSVAMEGEHLNSENSYDEVSLLKELARRQAIRDEMLQALFRPVKRCSDAQSDCAHRKKDGQCGSEKVKNVYCKKTCGGC |
|  |  | R.CSDAQSDCAHRK.K |  |  |
|  |  | K.NVYCKKTCGGC |  |  |
| c47813_g1_i1 | Unknown C6 | R.ELAEEELIAKR.V | R.CELSSSLSDADKK.G [93.4] | MKVAVKIALLFAVLMIVAEARSELAEEELIAKRIFDVLRDAACSGRCELSSSLSDADKKGRCRTIRNCWYPKYRCCT |
|  |  | R.DAACSGRCEL.S |  |  |
|  |  | R.DAACSGRCELS.S |  |  |
|  |  | R.DAACSGRCELSSSL.S |  |  |
|  |  | R.DAACSGRCELSSSLSDADK.K |  |  |
|  |  | R.DAACSGRCELSSSLSDADKKGR.C |  |  |
|  |  | R.NCWYPKY.R |  |  |
| c47813_g1_i2 | Unknown C6 | R.ELAEEELIAKR.V |  | MKVAVKIALLFAVLMIVAEARRELAEEELIAKRVFDVLRDSGCTGYCKLASKLSAAEKSGKCRTIRNCGYPKYRCCT |
|  |  | R.DSGCTGYCK.L |  |  |
|  |  | R.DSGCTGYCKL.A |  |  |
|  |  | R.DSGCTGYCKLA.S |  |  |
|  |  | R.DSGCTGYCKLASK.L |  |  |
|  |  | R.DSGCTGYCKLASKL.S |  |  |
|  |  | R.DSGCTGYCKLASKLSAAEK.S |  |  |
| c49643_g1_i1 | Unknown C8 | K.CGPCEIVNSHNY.C | Q.KYIDGSCK.T [90.1] | MKAILVFLVIAALTASSTAKPKPAIDFKKICFGRCQKYIDGSCKTDEACMAKIKAMSQKKCGPCEIVNSHNYCIPIANCQ |
|  |  | C.EIVNSHNYCIPIANCQ | K.YIDGSCK.T [87.6] |  |
|  |  | C.GPCEIVNSHNYCIPIAN.C | K.TDEACMAK.I [72.6] |  |
|  |  | S.HNYCIPIANCQ |  |  |
|  |  | Y.IDGSCKTDEACMAK.I |  |  |
|  |  | K.KCGPCEIVNSHNYCIPIANCQ |  |  |
|  |  | V.NSHNYCIPIANCQ |  |  |
|  |  | M.SQKKCGPCEIVNSHNYCIPIANCQ |  |  |
| c51674_g1_i3 | Knottin-like (ICK) | K.CVNVGSFSGPEYK.C |  | MKLALALVFLAITCMLSTDARNIWSFLDDYNDEIEKKGSCIPLGATGCEANNSKCCRKGDPYTGTLRKCVNVGSFSGPEYKCMEA |
|  |  | R.KCVNVGSFSGPEYK.C |  |  |
|  |  | R.KCVNVGSFSGPEYKCMEA |  |  |
|  |  | N.VGSFSGPEYK.C |  |  |
|  |  | C.VNVGSFSGPEYK.C |  |  |
| c52694_g1_i1 | KTx type I (ShK-like) | R.AECKDNLGTAECER.K | R.AECKDNLGTAECERK.K [94.4] | MKTQLLVVLLLCVLCSLSVAMEGEHLNNQNYDEASLLEILSRFEKRAECKDNLGTAECERKKGNCDSSMKYRLVNCRKTCGTCS |
|  |  | R.AECKDNLGTAECERK.K |  |  |
| c53934_g1_i2 | Unknown C6 | R.AGWQCSNGK.C | R.AGWQCSNGKCVP [63.6] | MMSTKILLVCLVVMFSVSCYATSVPETLRQLALDLEDPAASCMGKPCAYDRHCTSCRAGWQCSNGKCVP |
|  |  | R.AGWQCSNGKCVP |  |  |
|  |  | A.SCMGKPCAY.D |  |  |
| c56939_g1_i1 | Phospholipase A2-like (C10) | K.CPTIPPFK.E | R.FLDMEAEHSPCFNLVK.K [74.6] | MAKYLVIVAALIGVALANPTANPTAFPGTSWCGEDADNATMKCCQEHAKCPTIPPFKERYEIYNKLPVSIYECSCESRFYFCLKDAPLKKSLHRFLDMEAEHSPCFNLVKKHVCLATSWLDWFGFGCQKYGDVLVGEINWMKNLLKMFIPKKKQ |
|  |  | K.CPTIPPFKER.Y | R.YEIYNK.L [64.8] |  |
|  |  | R.YEIYNKLPVSIY.E |  |  |
|  |  | R.YEIYNKLPVSIYECSCESR.F |  |  |
| c56806_g1_i1 | Phospholipase A2 | K.CQMEVCK.C | Y.GIPVDPIDECCK.T [89.4] | MKVLQMFFCVILLCVTSVLVEAKSTTKGDETASKRNFAQFAAMTYHTTHRWPKKYVGYGCYCGLGGYGIPVDPIDECCKTHDACYKKVEDSGICSYSWAIYLTIYKRKGGAECSEDNEKCQMEVCKCDSVAAKCLGKYKDIFNEKYAGYDKKGKCDPSFTLS |
|  |  | K.GKCDPSF.T | K.GKCDPSFTLS [75.9] |  |
|  |  | K.KVEDSGICSY.S |  |  |
|  |  | K.KYVGYGCY.C |  |  |
|  |  | K.KYVGYGCYC.G |  |  |
|  |  | K.YAGYDKK.G |  |  |
|  |  | K.YKDIFNEK.Y |  |  |
|  |  | Y.GIPVDPIDECCK.T |  |  |
| c89724_g1_i1 | Unknown C6 | R.CLNVGQKC.T | R.CLNVGQK.C [60.1] | MKSFYGILCVAVLMMFHLEMSESRSNFQMLLEDPDYLSLEKRCLNVGQKCTPGNNKCCHTYICSSLDSKCFYR |
|  |  | R.CLNVGQKCTPGNNK.C |  |  |
|  |  | Y.ICSSLDSKCFYR |  |  |
|  |  | S.SLDSKCFYR |  |  |
|  |  | H.TYICSSLDSK.C |  |  |
|  |  | H.TYICSSLDSKCFY.R |  |  |
|  |  | T.YICSSLDSKCFY.R |  |  |
| c113611_g1_i1 | KTx type I (ShK) | H.CAVRLEL.G | R.LELGHCGPN.G [91.6] | MKYQLVLLWALVAFCCLSMEAQARRDEIMMKSLEELLRPQKRCEDIDAHCAVRLELGHCGPNGKAGIKEKYCRKTCGDC |
|  |  | R.CEDIDAHC.A |  |  |
|  |  | R.CEDIDAHCAVR.L |  |  |
|  |  | R.LELGHCGPNGK.A |  |  |

Supplementary Table 3: Comparison of toxin candidates identified in the transcriptome, and secreted and acontia proteome for *Calliactis polypus*. Acontia data derived from HL Smith et al., 2023. N.B. Unknown (C10) used to denote structural variants of one ShK-like and one PLA2-like toxin candidates.

| Functional Category | Toxin Family | Toxin Subtype | Transcriptome copy number | Secreted Proteome copy number | Acontia Proteome copy number |
| --- | --- | --- | --- | --- | --- |
| Enzyme | Lectin | C-Type | 4 |  |  |
| Enzyme | Lectin | Ficolin | 2 |  | 1 |
| Enzyme | Lipase | AB hydrolase | 2 |  |  |
| Enzyme | Lipase | Phospholipase A2 (PLA2) | 4 | 1 | 1 |
| Enzyme | Lipase | Type B carboxylesterase | 1 |  |  |
| Enzyme | Metalloprotease | Disintegrin and metalloprotease | 2 |  | 2 |
| Enzyme | Metalloprotease | Peptidase M12A | 8 |  | 2 |
| Enzyme | Protease | Multicopper oxidase | 4 |  | 1 |
| Neurotoxin | Potassium channel toxin | Kazal | 2 |  |  |
| Neurotoxin | Potassium channel toxin | Type I (ShK) | 4 | 3 | 1 |
| Neurotoxin | Potassium channel toxin | Type II (venom kunitz) | 5 |  |  |
| Neurotoxin | Potassium channel toxin | Type III | 1 |  | 1 |
| Neurotoxin | Sodium channel toxin | Calitoxin | 1 | 1 | 1 |
| Neurotoxin | Sodium channel toxin | Sea anemone sodium channel toxin | 2 |  | 1 |
| Neurotoxin | Sodium channel toxin | Type I | 1 |  | 1 |
| Neurotoxin | Structural class peptide | Sea anemone type 8 | 6 |  | 1 |
| Neurotoxin | Structural class peptide | Sea anemone type 9 | 1 | 1 |  |
| Unknown | Unknown | Unknown (C6) | 6 | 6 |  |
| Unknown | Unknown | Unknown (C8) | 1 | 1 |  |
| Unknown | Unknown | Unknown (C10) | 2 | 2 | 1 |
| Unknown | Unknown | Unknown 12C (HL Smith et al., 2023) | 1 |  | 1 |
| Unknown | Unknown | Cephalotoxin | 1 |  |  |
| Unknown | Unknown | VP302 | 4 |  |  |
|  |  | **Total** | **65** | **15 (23.07692%)** | **15** |

Supplementary Table 4: Comparison of transcript copy numbers for the sea anemone structural class 9 (c44591), NaTx type IV (calitoxin; c40761) and NaTx type I (c50240) candidates identified in the secreted venom of *Calliactis polypus*. N.B. All five domain structures for the SA9 sequences have been combined into a single column.

| Superfamily | Species | Transcript Copy number | | | Total sequences per species |
| --- | --- | --- | --- | --- | --- |
|  |  | *c44591* | *c40761* | *c50240* |  |
| Actinioidea | *Actinia tenebrosa* | 1 |  | 1 | 2 |
| Actinioidea | *Actinodendron plumosum* |  |  |  | 0 |
| Actinioidea | *Anemonia sulcata* |  |  |  | 0 |
| Actinioidea | *Anthopleura buddemeieri* | 1 |  | 1 | 2 |
| Actinioidea | *Aulactinia veratra* |  |  |  | 0 |
| Actinioidea | *Condylactis gigantea* |  |  |  | 0 |
| Actinioidea | *Entacmaea quadricolor* | 2 |  |  | 2 |
| Actinioidea | *Heterodactyla hemprichii* |  |  |  | 0 |
| Actinioidea | *Megalactis griffithsi* |  |  |  | 0 |
| Actinioidea | *Stichodactyla mertensii* | 2 |  |  | 2 |
| Edwardsioidea | *Edwardsiella carnea* |  |  |  | 0 |
| Edwardsioidea | *Nematostella vectensis* |  |  |  | 0 |
| Metridioidea | *Alvinactis* sp. | 4 |  |  | 4 |
| Metridioidea | *Calliactis polypus* | 2 | 1 | 2 | 5 |
| Metridioidea | *Exaiptasia diaphana* |  |  |  | 0 |
| Metridioidea | *Metridium senile* | 5 | 1 |  | 6 |
| Metridioidea | *Nemanthus annamensis* | 2 | 1 | 3 | 6 |
| Metridioidea | *Telmatactis stephensoni* | 1 |  |  | 1 |
| Metridioidea | *Triactis producta* |  |  |  | 0 |
| Total sequences per transcript | | 20 | 3 | 7 |  |

Supplementary Table 5: Comparison of transcript copy numbers for the ShK and ShK-like candidates identified in the secreted venom of *Calliactis polypus*.

| Superfamily | Species | Transcript Copy number | | | | Total sequences per species |
| --- | --- | --- | --- | --- | --- | --- |
|  |  | *c32422* | *c45616* | *c52694* | *c113611* |  |
| Actinioidea | *Actinia tenebrosa* | 1 |  | 1 |  | 2 |
| Actinioidea | *Actinodendron plumosum* |  |  |  |  | 0 |
| Actinioidea | *Anemonia sulcata* |  |  | 1 |  | 1 |
| Actinioidea | *Anthopleura buddemeieri* |  |  | 1 |  | 1 |
| Actinioidea | *Aulactinia veratra* |  |  |  |  | 0 |
| Actinioidea | *Condylactis gigantea* |  |  |  |  | 0 |
| Actinioidea | *Entacmaea quadricolor* |  |  |  |  | 0 |
| Actinioidea | *Heterodactyla hemprichii* |  |  |  |  | 0 |
| Actinioidea | *Megalactis griffithsi* |  |  |  |  | 0 |
| Actinioidea | *Stichodactyla mertensii* |  |  | 2 |  | 2 |
| Edwardsioidea | *Edwardsiella carnea* |  |  |  |  | 0 |
| Edwardsioidea | *Nematostella vectensis* |  |  |  |  | 0 |
| Metridioidea | *Alvinactis* sp. | 3 |  | 1 |  | 4 |
| Metridioidea | *Calliactis polypus* | 2 | 1 | 1 | 3 | 7 |
| Metridioidea | *Exaiptasia diaphana* | 3 |  |  |  | 3 |
| Metridioidea | *Metridium senile* | 1 |  |  |  | 1 |
| Metridioidea | *Nemanthus annamensis* |  |  |  |  | 0 |
| Metridioidea | *Telmatactis stephensoni* | 2 |  | 1 |  | 3 |
| Metridioidea | *Triactis producta* |  |  |  |  | 0 |
| Total sequences per transcript | | 12 | 1 | 8 | 3 |  |

Supplementary Table 6: Fixed Effects Likelihood analysis for the predicted mature peptide of the uncharacterized toxin candidate, Transcript c32422_g1_i1, using the DataMonkey 2.0 server (http://www.datamonkey.org/). Analysis was performed using default settings for 100 iterations with Syn Rate Variation and Profile Likelihood for each site applied. Negative selection reported for all codons with statistical significance (p-value < 0.05), with all other sites for neutral selection and insignificant values (p-value > 0.05) not reported.

| Codon | AA | Alternative AA | dN/dS | codon | alpha | beta | alpha=beta | LRT | p-value | Total branch length | dN/dS LB | dN/dS MLE | dN/dS UB | p-asmp |
| --- | --- | --- | --- | --- | --- | --- | --- | --- | --- | --- | --- | --- | --- | --- |
| 1 | D |  | 0 | 1 | 2.124 | 0 | 0.064 | 17.65 | 0.0099 | 1.084 | 0 | 0 | 0.019 | 0 |
| 3 | L |  | 0.043824701 | 3 | 0.753 | 0.033 | 0.179 | 10.191 | 0.0099 | 3.031 | 0.002 | 0.044 | 0.202 | 0.0014 |
| 4 | S |  | 0.138075314 | 4 | 0.478 | 0.066 | 0.104 | 3.024 | 0.0495 | 1.769 | 0.033 | 0.138 | 0.383 | 0.0821 |
| 7 | S | H | 0.0422852 | 7 | 2.223 | 0.094 | 0.159 | 6.747 | 0.0198 | 2.7 | 0.013 | 0.042 | 0.107 | 0.0094 |
| 8 | C |  | 0 | 8 | 1.963 | 0 | 0.085 | 12.982 | 0.0099 | 1.442 | 0 | 0 | 0.031 | 0.0003 |
| 17 | C |  | 0 | 17 | 0.511 | 0 | 0.055 | 8.287 | 0.0099 | 0.924 | 0 | 0 | 0.116 | 0.004 |
| 19 | Y | F/L | 0.001027141 | 19 | 147.01 | 0.151 | 0.278 | 6.481 | 0.0099 | 4.706 | 0 | 0.001 | 0.003 | 0.0109 |
| 26 | L |  | 0.066666667 | 26 | 1.59 | 0.106 | 0.295 | 9.162 | 0.0099 | 5.001 | 0.016 | 0.066 | 0.189 | 0.0025 |
| 27 | C |  | 0 | 27 | 1.353 | 0 | 0.084 | 13.139 | 0.0099 | 1.431 | 0 | 0 | 0.044 | 0.0003 |
| 29 | A | T | 0.17816092 | 29 | 0.696 | 0.124 | 0.205 | 4.182 | 0.0396 | 3.467 | 0.056 | 0.178 | 0.423 | 0.0408 |
| 30 | T |  | 0.1875 | 30 | 0.592 | 0.111 | 0.2 | 4.174 | 0.0396 | 3.388 | 0.056 | 0.187 | 0.474 | 0.041 |
| 31 | C |  | 0 | 31 | 0.376 | 0 | 0.027 | 4.476 | 0.0396 | 0.456 | 0 | 0 | 0.159 | 0.0344 |
| 44 | C |  | 0 | 44 | 0.491 | 0 | 0.055 | 8.246 | 0.0099 | 0.925 | 0 | 0 | 0.121 | 0.0041 |
| 48 | L |  | 0.011916922 | 48 | 2.937 | 0.035 | 0.173 | 5.926 | 0.0198 | 2.928 | 0.001 | 0.012 | 0.056 | 0.0149 |
| 49 | S |  | 0.070621469 | 49 | 1.062 | 0.075 | 0.228 | 7.099 | 0.0198 | 3.864 | 0.011 | 0.071 | 0.231 | 0.0077 |
| 52 | S |  | 0.016367888 | 52 | 1.283 | 0.021 | 0.082 | 10.651 | 0.0099 | 1.385 | 0.001 | 0.017 | 0.075 | 0.0011 |
| 53 | C |  | 0 | 53 | 1.857 | 0 | 0.089 | 14.961 | 0.0099 | 1.505 | 0 | 0 | 0.032 | 0.0001 |
| 56 | K |  | 0.034662577 | 56 | 3.26 | 0.113 | 0.281 | 7.935 | 0.0099 | 4.769 | 0.008 | 0.035 | 0.1 | 0.0048 |
| 62 | C |  | 0 | 62 | 0.447 | 0 | 0.055 | 7.861 | 0.0099 | 0.931 | 0 | 0 | 0.136 | 0.0051 |
| 72 | C |  | 0 | 72 | 0.842 | 0 | 0.084 | 12.486 | 0.0099 | 1.429 | 0 | 0 | 0.071 | 0.0004 |
| 75 | T |  | 0.038858049 | 75 | 1.261 | 0.049 | 0.123 | 7.318 | 0.0099 | 2.086 | 0.006 | 0.039 | 0.125 | 0.0068 |
| 76 | C |  | 0 | 76 | 1.06 | 0 | 0.084 | 12.852 | 0.0099 | 1.428 | 0 | 0 | 0.056 | 0.0003 |
| 79 | C |  | 0 | 79 | 1.5 | 0 | 0.056 | 10.791 | 0.0099 | 0.947 | 0 | 0 | 0.04 | 0.001 |

Supplementary Table 7: Comparison of transcript copy numbers for the functional uncharacterized (Unknown) candidates identified in the secreted venom of *Calliactis polypus*.

| Superfamily | Species | Transcript Copy number | | | | | | Total sequences per species |
| --- | --- | --- | --- | --- | --- | --- | --- | --- |
|  |  | *c1610* | *c47813* | *c49643* | *c51674* | *c53934* | *c89724* |  |
| Actinioidea | *Actinia tenebrosa* |  |  |  |  |  |  | 0 |
| Actinioidea | *Actinodendron plumosum* |  |  |  |  |  |  | 0 |
| Actinioidea | *Anemonia sulcata* | 1 |  |  |  |  |  | 1 |
| Actinioidea | *Anthopleura buddemeieri* | 2 |  |  |  |  |  | 2 |
| Actinioidea | *Aulactinia veratra* |  |  |  |  |  |  | 0 |
| Actinioidea | *Condylactis gigantea* |  |  |  |  |  |  | 0 |
| Actinioidea | *Entacmaea quadricolor* |  |  |  |  |  |  | 0 |
| Actinioidea | *Heterodactyla hemprichii* |  |  |  |  |  |  | 0 |
| Actinioidea | *Megalactis griffithsi* |  |  |  |  |  |  | 0 |
| Actinioidea | *Stichodactyla mertensii* |  |  |  |  |  |  | 0 |
| Edwardsioidea | *Edwardsiella carnea* |  |  |  |  |  |  | 0 |
| Edwardsioidea | *Nematostella vectensis* |  |  |  |  |  |  | 0 |
| Metridioidea | *Alvinactis* sp. |  |  |  |  |  |  | 0 |
| Metridioidea | *Calliactis polypus* | 1 | 4 | 1 | 1 | 3 | 4 | 14 |
| Metridioidea | *Exaiptasia diaphana* | 1 |  |  |  |  |  | 1 |
| Metridioidea | *Metridium senile* | 1 |  |  |  |  | 1 | 2 |
| Metridioidea | *Nemanthus annamensis* |  | 2 |  |  | 1 | 1 | 4 |
| Metridioidea | *Telmatactis stephensoni* |  | 1 |  |  |  |  | 1 |
| Metridioidea | *Triactis producta* |  |  |  |  |  |  | 0 |
| Total sequences per transcript | | 6 | 7 | 1 | 1 | 4 | 6 |  |
